# Assessing the Impact of Prophylactic Eculizumab on Renal Graft Survival in Atypical Hemolytic Uremic Syndrome

**DOI:** 10.1097/TP.0000000000004355

**Published:** 2023-03-31

**Authors:** Emily K. Glover, Kate Smith-Jackson, Vicky Brocklebank, Valerie Wilson, Patrick R. Walsh, Emma K. Montgomery, Edwin K.S. Wong, Sally Johnson, Michal Malina, David Kavanagh, Neil S. Sheerin

**Affiliations:** 1 National Renal Complement Therapeutics Centre, Newcastle upon Tyne Hospitals NHS Foundation Trust, Newcastle Upon Tyne, UK.; 2 Translational and Clinical Research Institute, Newcastle University, Newcastle Upon Tyne, UK.; 3 NIHR Newcastle Biomedical Research Centre, Biomedical Research Building, Campus for Ageing and Vitality, Newcastle upon Tyne, UK.; 4 Great North Children’s Hospital, Newcastle Upon Tyne Hospitals NHS Foundation Trust, Newcastle upon Tyne, UK.

## Abstract

Atypical hemolytic uremic syndrome (aHUS) is a rare cause of end-stage kidney disease and associated with poor outcomes after kidney transplantation from early disease recurrence. Prophylactic eculizumab treatment at the time of transplantation is used in selected patients with aHUS. We report a retrospective case note review describing transplant outcomes in patients with aHUS transplanted between 1978 and 2017, including those patients treated with eculizumab.

**Methods:**

The National Renal Complement Therapeutics Centre database identified 118 kidney transplants in 86 recipients who had a confirmed diagnosis of aHUS. Thirty-eight kidney transplants were performed in 38 recipients who received prophylactic eculizumab. The cohort not treated with eculizumab comprised 80 transplants in 60 recipients and was refined to produce a comparable cohort of 33 transplants in 32 medium and high-risk recipients implanted since 2002. Complement pathway genetic screening was performed. Graft survival was censored for graft function at last follow-up or patient death. Graft survival without eculizumab treatment is described by complement defect status and by Kidney Disease: Improving Global Outcomes risk stratification.

**Results:**

Prophylactic eculizumab treatment improved renal allograft survival (*P* = 0.006) in medium and high-risk recipients with 1-y survival of 97% versus 64% in untreated patients. Our data supports the risk stratification advised by Kidney Disease: Improving Global Outcomes.

**Conclusions.:**

Prophylactic eculizumab treatment dramatically improves graft survival making transplantation a viable therapeutic option in aHUS.

## Graphical abstract

**Figure:**
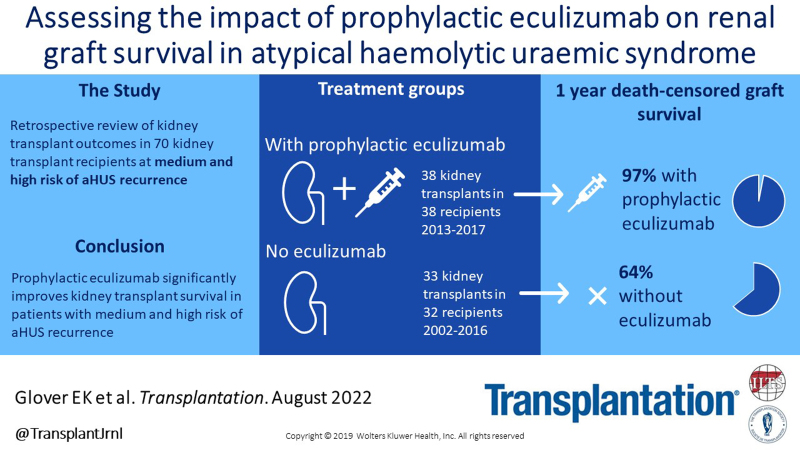

## INTRODUCTION

Hemolytic uremic syndrome (HUS) is a type of thrombotic microangiopathy (TMA) and describes a group of rare, life-threatening diseases characteristically presenting with microangiopathic hemolytic anemia, acute kidney injury, and thrombocytopenia coupled with histological evidence of endothelial injury.^1^ The kidney is the most vulnerable organ but other organ involvement is recognized. HUS is classified according to its etiology: Shiga-toxin producing *Escherichia coli* (accounting for 90% of cases), secondary HUS when there is an identifiable cause, and atypical HUS (aHUS).^2^ aHUS encompasses complement and noncomplement mediated causes.^3^

The essential role of the complement alternative pathway in the pathogenesis of aHUS is well established; through genomic studies^4^ and with the effective use of complement inhibition as treatment.^5,6^ Approximately, 60% to 70% of patients with aHUS have inherited or acquired dysregulation of the complement alternative pathway.^3^ Loss of function genetic variants are identified in genes encoding complement regulatory proteins; complement factor H (*CFH*), complement factor I (*CFI*), membrane cofactor protein (*CD46*), and gain of function variants in genes encoding activating proteins; *C3* and complement factor B (*CFB*).^2,7^ For people with a disease-associated genetic variant, clinical manifestations usually develop when their genetic susceptibility is challenged by environmental triggers.

aHUS has an incidence of 0.5 per million.^3^ Until recently, treatment relied upon plasma exchange and supportive therapies, with a mortality risk in the acute phase of 10% to15% and approximately 50% of patients progressing to end-stage kidney disease (ESKD) with their first episode.^8,9^ Despite the high rate of ESKD, these patients were previously precluded from renal transplantation by the high risk of recurrent disease, the exception being for a dual liver-kidney transplant in those patients with pathogenic variants in proteins produced in the liver^10^ or for patients with pathogenic variants in *CD46*. Disease recurrence predominantly occurred in the first year and resulted in graft loss for up to 90%.^11,12^

Eculizumab, a monoclonal antibody targeting C5, blocks complement terminal pathway activity. It has revolutionized renal outcomes in aHUS for disease in both native and transplanted kidneys.^13-15^ In native kidneys, an advantage has been demonstrated for early treatment.^13^ As most posttransplant aHUS recurrence occurs within the first year,^8,11^ prophylactic eculizumab therapy initiated at transplantation has been proposed to prevent disease recurrence and associated graft injury. Cause of complement dysregulation and previous transplant history was proposed to guide recurrence risk stratification.^8,16^ In 2016, Kidney Disease: Improving Global Outcomes (KDIGO) advised prophylactic therapy for transplant recipients considered at medium or high risk of disease recurrence.^3^ Data from France^17^ support this strategy for improving transplant outcomes in aHUS.

In the UK, patients with suspected aHUS are referred to a single-specialist center for further investigation and access to eculizumab.^18^ We conducted a retrospective study of renal transplant recipients with a known diagnosis of aHUS, to assess the efficacy of prophylactic eculizumab treatment compared with patients with similar defects in complement regulation who were transplanted in the pre-eculizumab era.

## MATERIALS AND METHODS

### Study Groups

The National Renal Complement Therapeutics Centre, Newcastle upon Tyne, UK (NRCTC; http://www.atypicalhus.co.uk) performs genetic screening for patients with complement mediated kidney diseases. Patients with ESKD attributed to aHUS were identified from the National Renal Complement Therapeutics Centre cohort through the combination of clinical features, renal histology, and exclusion of other causes of TMA (**Table S1, SDC**, http://links.lww.com/TP/C577). Screening for complement pathway abnormalities was undertaken as detailed below.

**FIGURE 1.**
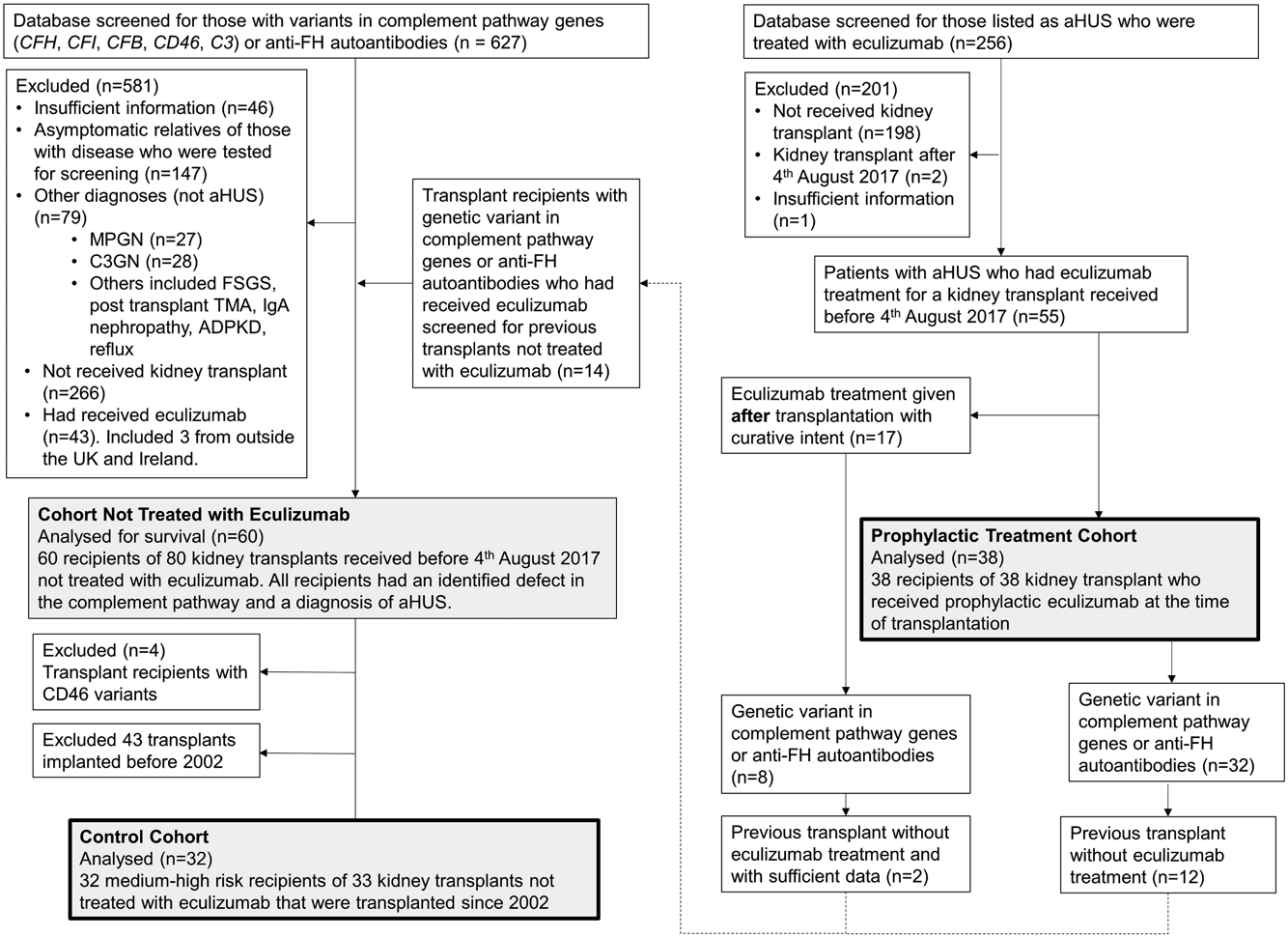
Consort diagram. Identification of control and prophylactic treatment cohorts and historic cohort not treated with eculizumab, from the National Renal Complement Therapeutics Centre database.

The prophylactic treatment cohort and cohort not treated with eculizumab were identified as detailed in Figure 1. Those not treated with eculizumab received a single organ kidney transplant without eculizumab treatment at any stage during the transplant procedure.

The prophylactic treatment cohort comprised patients who received prophylactic eculizumab to prevent aHUS recurrence in their renal transplant. Since 2013 eculizumab has been available for the treatment of aHUS in the UK. Patients with aHUS being considered for renal transplantation were stratified by risk of disease recurrence^3,16^ with those at medium or high risk being approved for prophylactic eculizumab. The protocol, for prophylactic eculizumab treatment was a single dose of 900 mg just before surgery. Adult patients received 3 further weekly doses of 900 mg, then 1200 mg after a further week before continuing on 1200 mg every 2 wk. A further dose of eculizumab was advised if blood loss requiring administration of Fresh Frozen Plasma or equivalent occurred^19^ and a dose increase was made if there was breakthrough complement activity. In pediatric patients dose was adjusted by weight as detailed in Summary of Product Characteristics.^20^

Patients on eculizumab are at increased risk of *Neisseria meningitides* infection.^20^ Vaccination against serotypes ACWY and serotype B (available in the UK since 2015) was recommended before starting eculizumab^21,22^ and prophylactic antibiotics were advised for the duration of eculizumab therapy.^22^

As the prophylactic treatment cohort consisted only of recipients at medium or high risk of disease recurrence, a subgroup of comparable recipients in the untreated cohort was generated. This excluded recipients with rare genetic variants in *CD46* due to their recognized low risk of disease recurrence.^3^ Given the advances in immunosuppression over the period of these transplants (1978-2016) and improved graft survival,^23^ we assessed outcome in transplants stratified by year of transplantation. DCD kidney transplant survival has improved most dramatically over the years with NHSBT data giving 5-y graft survival of 73% for those implanted in 1999–2001,^24^ compared with 86% for grafts implanted in 2002–2004 or later.^25,26^ For this reason, transplants implanted before 2002 were compared with those from 2002 onward. The lower survival in the pre-2002 transplants was consistent with an era effect (**Figure S1, SDC**, http://links.lww.com/TP/C577). We therefore included only transplants received since 2002 in the control cohort to be compared with prophylactic treatment.

To assess the impact of recipient complement defects on graft outcome, transplants in the cohort not treated with eculizumab were categorized firstly by the affected gene if a pathogenic variant in *C3*, *CFB*, *CFH*, *CFI*, or *CD46* was detected. Variants of uncertain significance (VUSs) in any of these genes were grouped together. When assigning transplants in factor H autoantibody (anti-FH) positive recipients to an analysis group, those with isolated anti-FH autoantibodies or concomitant VUS were assigned to the anti-FH group, whereas those with pathogenic variants went by the affected gene.

All transplants were performed before August 4, 2017 to allow 36 mo follow-up.

### Data Collection

Available medical notes were reviewed for all groups. Transplant recipient sex, age at transplantation, year of transplantation, graft outcome, and cause of death with functioning graft were collected. Previous kidney transplant history was collected when available. Further data points collected when available for control and prophylactic treatment cohorts are detailed in **Table S2, SDC**, http://links.lww.com/TP/C577. Graft survival was reported at patient death with a functioning graft or last follow-up.

### Genetic Analysis

Variant screening of *CFH*, *CFI*, *CFB*, *C3*, and *CD46* was undertaken using Sanger sequencing, as previously described.^27-31^ Screening for chromosomal rearrangements affecting *CFH*, *CFHR1*, *CFHR2*, *CFHR3*, *CFHR4*, *CFHR5*, *CFI*, and *CD46* was undertaken using multiplex ligation-dependent probe amplification, as previously described.^32,33^ To assess for genetic abnormalities in noncomplement genes associated with aHUS including *DGKE*,^34^ *MMACHC*, *VTN*, *PLG*, *THBD*, and *IFN2*^35^ Sanger sequencing was performed (**Tables S3A–C, SDC**, http://links.lww.com/TP/C577) in selected patients.

Rare genetic variants were evaluated using Alamut Visual 2.10 (2017 Interactive Biosoftware). Variants were classified in 2019 according to American College of Medical Genetics and Genomics guidelines^36^ with refinement developed by Sequence Variant Interpretation Working Group.^37^

### Factor H Autoantibodies

The consensus ELISA assay was performed to detect anti-FH autoantibodies, as previously described.^38^ The role of anti-FH autoantibodies in aHUS was recognized in 2005^39^ so testing was not performed in every case.

### Statistical Analysis

Renal graft survival, analyzed with Kaplan-Meier curves, was censored for patient death with a functioning graft and for functioning graft at last follow. Log-rank test assessed the difference between survival of 2 groups.

Group characteristics were compared using T-test for continuous variables (age) and Fisher exact test or Chi-squared tests for categorical variables as determined by expected counts. Bonferroni adjustment was used for multiple pairwise comparisons. Mann-Whitney U test compared median time with presentation, follow-up, and year of transplantation.

Analysis was performed using Rstudio Team (2021). RStudio: Integrated Development Environment for R. R Studio, PBC, Boston, MA URL, http://www.rstudio.com/. *P* < 0.05 was considered statistically significant.

### Ethics Statement

This study was exempt from NHS Research Ethics Review.

## RESULTS

### Impact of Prophylactic Eculizumab Treatment

To assess the impact of prophylactic eculizumab treatment we compared graft survival of transplants performed with eculizumab treatment with transplants performed after 2002 in recipients at medium and high risk of recurrence who had not received eculizumab (control cohort). Grafts implanted between 2002 and 2017 were considered comparable as there has been little change in kidney graft survival in the UK during this period.^25,26^

#### Demographics and Complement Defects

Recipient demographics, complement defect status, and transplant details are given in Table 2. There were more children in the control versus treatment cohort (27% versus 7.9%, *P* = 0.064). Thirteen of the 14 in the prophylactic treatment group that were being retransplanted had experienced previous graft loss from posttransplant TMA (**Supplemental Data Sets, SDC**, http://links.lww.com/TP/C576).

**TABLE 1.** Criteria used to determine risk of recurrence of aHUS in transplanted kidney^3^

High risk of recurrence	Medium risk of recurrence	Low risk of recurrence
• Mutations in Factor H or gene rearrangements involving Factor H or Factor H related proteins • Gain of function mutations in Factor B or C3 • Loss previous transplant due to recurrent aHUS	• No identified mutation or autoantibody • Mutations in factor I • Mutation of uncertain functional significance • Detectable autoantibodies against factor H	• Mutation in membrane cofactor protein CD46 • Previous autoantibody positivity

aHUS, atypical hemolytic uremic syndrome.

**TABLE 2.** Demographics, baseline characteristics, and immunosuppressive treatments of control and prophylactic eculizumab treatment cohorts

	Control cohort	Prophylactic treatment cohort
Kidney transplants	33	38
Transplant recipients	32	38
Demographics		
Mean age in y at transplantation (SD)	28.3 (±15.6)**	38.7 (±13.6)
Children (<16 y old) (% of those with available data)	9 (27.3)	3 (7.9)
Female recipients (% of transplants)	25 (75.8)	25 (65.8)
Median y of transplantation (range)	2007 (2002-2016) ***	2015 (2013-2017)
Recipient complement defect status (% of transplants)		
Nil	0	6 (15.7)
VUS	10 (30.3)	6 (15.7)
Pathological variant	21 (63.6)	24 (63.2)
Complement factor B	0	0
Complement factor H (CFH)	17 (51.5)	16 (42.1)
Complement factor I (CFI)	2 (6.1)	0
CFH + CFI	0	1 (2.6)
C3	2 (6.1)	7 (18.4)
Number tested for anti-FH	28 (87.5)	32 (84.2)
Isolated anti-FH detected	2	2
Anti-FH detected with genetic variant	5	1
Risk of aHUS recurrence		
Medium	14 (42.4)	12 (31.6)
High	19 (57.6)	26 (68.4)
Transplant details		
First transplants (%)	26 (78.8)	24 (63)
Live donor transplants (% of n)	n = 2210 (45.5)	n = 388 (21)
Median HLA mismatch (range)	not available	3 (0-6)n = 33
Plasma exchange		
Received posttransplant PLEX	7***(n = 11 with available data)	1
Immunosuppression		
Available data on induction immunosuppression	not available	n = 23
Basilixumab		19
Antithymocyte globulin		4
Alemtuzumab		0
Available data on maintenance immunosuppression	not available	n = 33
Tacrolimus + Mycophenolate mofetil + Prednisolone		28
Tacrolimus + Mycophenolate mofetil		1
Ciclosporin + Mycophenolate mofetil		0
Tacrolimus + Prednisolone		2
Tacrolimus + Azathioprine + Prednisolone		2
Meningococcal vaccine received before first D of eculizumab treatment		
ACWY serotype (n gives those with available data)	not applicable	32 (n = 33)
B serotype (n gives those with available data)	not applicable	26 (n = 32)

Data for kidney transplants received since 2002 at medium or high risk of recurrence of atypical hemolytic uremic syndrome (aHUS) who were not treated with eculizumab (control cohort) and kidney transplants treated with prophylactic eculizumab at the time of transplantation (prophylactic treatment cohort). No recipients had identified pathological variants in noncomplement pathway gene associated with aHUS. Whenever data are not available for complete group, number with available data is given (n).

anti-FH, factor H autoantibody; PLEX, plasma exchange; VUS, variant of uncertain significance.

*P < 0.05,

** *P* < 0.01,

*** *P* < 0.001 for difference compared with prophylactic treatment group.

Complement defects were similarly distributed within each group (Table 2). Pathogenic variants in *CFH* were the most common, in keeping with the literature and included point mutations, exon deletions, exon duplications, premature stop codons, and chromosomal rearrangements.^17,40^ Recipients classified as high risk of aHUS recurrence based on genetic screening results, autoantibody testing, and previous transplant history,^3^ predominated in both groups (58% in control versus 68% treatment cohort, *P* = 0.485).

#### Clinical Features

The prophylactic treatment cohort all had a clinical diagnosis of aHUS as cause for acute kidney injury and ultimately native ESKD. Twenty-five had a native kidney biopsy showing TMA. In the control cohort, 17 had an available kidney biopsy result of which 14 showed TMA (**Supplemental Data Sets, SDC**, http://links.lww.com/TP/C576).

#### Transplant Biopsy Results

In the control cohort, transplant kidney biopsy data were available for 17. Fourteen showed TMA. In the prophylactic eculizumab group, 18 kidney transplants were biopsied and 11 showed rejection. Two showed TMA (**Supplemental Data Sets, SDC**, http://links.lww.com/TP/C576) and were in recipients with pathological variants in *CFH*. C4d staining was negative in both cases. In 1 (#47) TMA was shown 4 d posttransplant with evidence of microangiopathic hemolytic anemia despite absent terminal pathway activity. She received plasma exchange, repeat eculizumab dosing and the graft continues to function. In the other (#118), initial transplant biopsy <3 mo posttransplant showed acute T cell mediated rejection but with endothelial swelling and loss of fenestrations on electron microscopy, consistent with TMA (no donor-specific antibodies detected). Subsequent biopsies did not demonstrate TMA until graft loss, 6-y posttransplant, when chronic TMA was identified. As there were concerns of incomplete complement blockade 6 mo posttransplant, the fortnightly dose was increased to 1500 mg, after which complement activity was fully blocked.

#### Graft Function

In the prophylactic treatment cohort, 27 had immediate graft function and the remaining 5 with available data had delayed graft function. In adults with available data mean (SE) 1-y creatinine was 1.71 (0.25) mg/dL (n = 28) and eGFR was 60.0 (5.3) ml/min/1.73 m^2^ (n = 21). In the 3 children (aged 9-11 y), mean (SE) creatinine at 1 y was 0.56 (0.29) mg/dL and eGFR was 126.8 (29.2) ml/min/1.73 m^2^.

#### Graft Outcome

**FIGURE 2.**
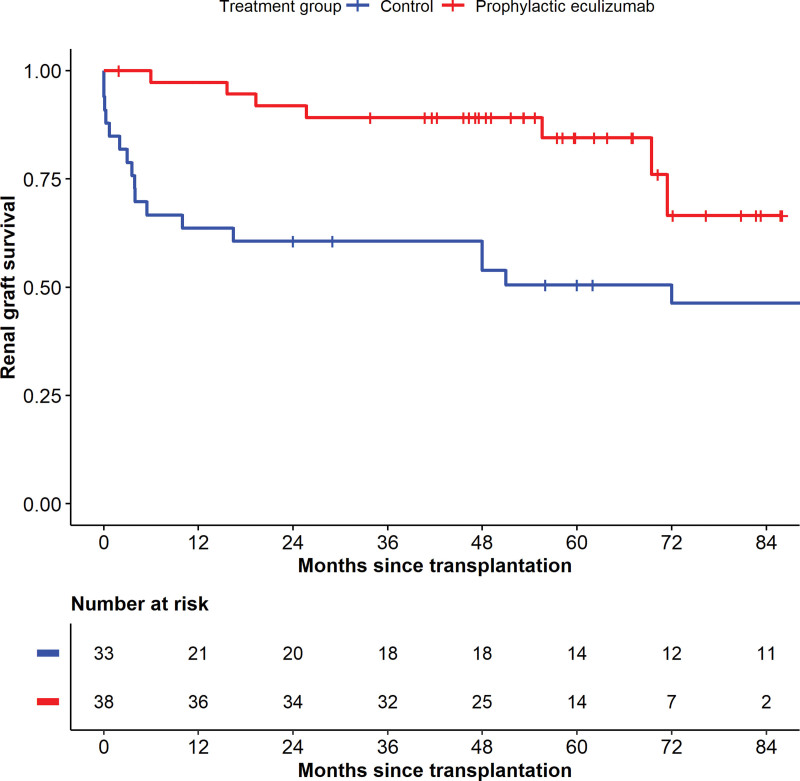
Death-censored renal graft survival with and without prophylactic eculizumab treatment. Kaplan-Meier analysis of renal graft survival for grafts received after 2002 in recipients at medium or high risk of recurrence of atypical hemolytic uremic syndrome. Those who received prophylactic eculizumab treatment from the time of transplantation compared with those who did not receive eculizumab treatment for the duration of the transplant (control). Numbers at risk in each group at 6 monthly time points are detailed below the graph. Log-rank *P* = 0.006.

Graft outcome is detailed in Table 3. A significantly higher percentage of transplants continued to function in the prophylactic eculizumab treatment cohort compared with the control cohort (81.6% versus 33.3%, *P* < 0.001). Death-censored graft survival was significantly better with prophylactic eculizumab treatment compared with controls (Figure 2, log-rank *P* = 0.006). In the control cohort 3-mo, 1-, and 3-y cumulative death-censored graft survival was 79%, 64%, and 61%, respectively. In those treated with prophylactic eculizumab, graft survival at the same time points was 100%, 97%, and 89% respectively. Fewer transplants were lost from posttransplant TMA in the treatment cohort compared with the control cohort (42.4% versus 2.6%, *P* < 0.001).

**Table 3.** Graft outcomes in those with atypical hemolytic uremic syndrome with and without prophylactic eculizumab treatment

Graft outcome	Control cohort (n = 33)	Prophylactic eculizumab cohort (n = 38)
Functioning (%)	11 (33.3)***	31 (81.6)
Failed (%)	22 (66.7)***	7 (18)
Cause of graft failure (% of transplants)		
Posttransplant TMA	14 (42.4)***	1 (2.6)
Rejection	5 (15.2)	4 (10.5)
Other	3 (9.1): graft thrombosis, nonviable infarcted kidney with primary nonfunction, transplant glomerulopathy	2 (5.3): meningococcal sepsis, immune complex mediated glomerulonephritis
Recipient death with functioning graft (% of those with functioning graft)	0	5 (16.1)
Median (range) follow-up time for those alive with functioning graft	7.9 y (2.0-16.0 y)	1.1 y (3.4–7.2 y)

Data for kidney transplants received since 2002 at medium or high risk of recurrence of atypical hemolytic uremic syndrome who were not treated with eculizumab (control) and kidney transplants treated with prophylactic eculizumab at the time of transplantation (prophylactic treatment cohort).

TMA, thrombotic microangiopathy.

*P < 0.05,

**P < 0.01,

*** *P* < 0.001 for difference in proportion of control cohort meeting this criteria compared with prophylactic treatment cohort. There was no difference in median follow-up between groups (*P* = 0.070).

One graft treated with prophylactic eculizumab failed following *Neisseria meningitidis B* bacteremia (#300). The recipient had not received B serotype vaccination or antibiotics for meningococcal prophylaxis. This was the only case of meningococcal disease recorded.

#### Deaths With a Functioning Graft

In the prophylactic eculizumab treatment cohort, 5 recipients died with a functioning graft, in 4 this was over 2 y posttransplant. One death was attributed to multiorgan failure associated with disseminated Candida and Herpes simplex virus infections (Supplemental Data Sets, **SDC**, http://links.lww.com/TP/C576). There were no other infection-related deaths. There were no deaths with a functioning transplant in the control cohort (*P* = 0.057).

#### Eculizumab Cessation

Eculizumab continued for the duration of renal graft function in all but 4 transplant recipients treated with prophylactic eculizumab and 2 of these continue to function (1 with *CFI* VUS, 1 without variants). In a recipient with no variant (#575), eculizumab was withdrawn at 6 mo and the graft failed from chronic rejection after 5 y. The other had a *CFHR1/CFH* hybrid (#176) and stopped after 8 mo due to declining function without evidence of aHUS recurrence. This graft failed at 15 mo with no evidence of TMA on biopsy (**Supplemental Data Sets, SDC**, http://links.lww.com/TP/C576).

### Impact of Complement Defect

#### Demographics and Complement Defects

Eighty kidney transplants in 60 recipients with aHUS were transplanted between 1978 and 2016 and not treated with eculizumab (**Supplemental Data Sets, SDC**, http://links.lww.com/TP/C576). The majority of recipients were female (60%). Mean (SD) age at transplantation was 25.6 (±13.6) y. Eighteen children were included.

Given the method of identification (Figure 1), all recipients had a complement pathway defect. *CFH* was the gene most frequently affected by pathological variants (n = 43). The distribution of complement defects is show in **Table S4, SDC**, http://links.lww.com/TP/C577.

#### Graft Survival

**FIGURE 3.**
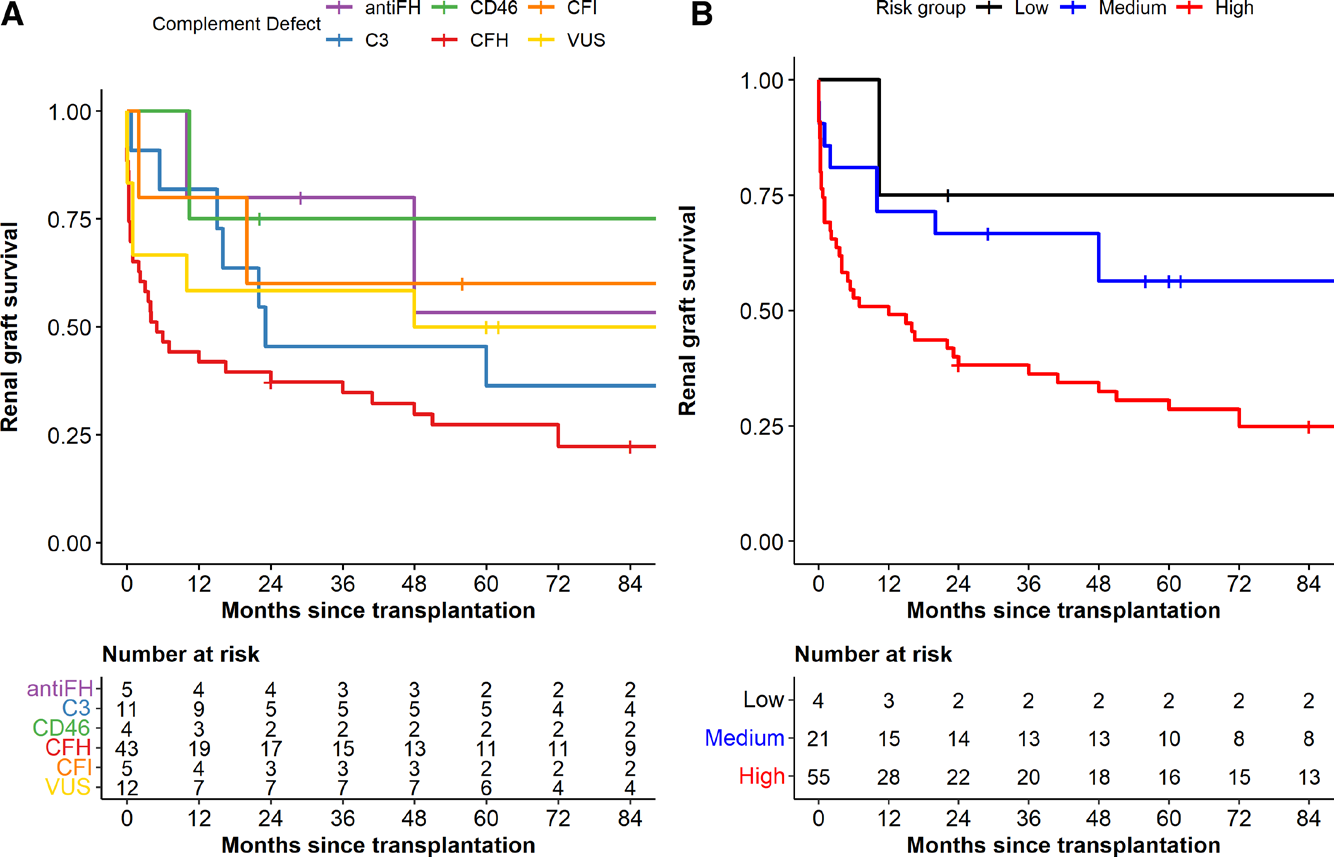
Death-censored renal graft survival without eculizumab treatment. Kaplan-Meier analysis of overall renal graft survival in recipients transplanted between 1978 and 2016 for atypical hemolytic uremic syndrome who did not receive eculizumab treatment with the transplant. Survival is censored for patient death with a functioning graft and for functioning graft at last follow-up. Numbers at risk in each group at 12 monthly time points are detailed below the graph. A. Graft survival by complement defect. Grafts grouped by presence of autoantibodies against Factor H (anti-FH), variant of uncertain significance (VUS) or pathogenic variant in complement factor I (CFI), complement factor H (CFH), membrane cofactor protein (CD46), or C3 in recipient. B. Graft survival by risk of relapse. Grafts grouped by low, medium, or high risk of atypical hemolytic syndrome recurrence (as stratified by KDIGO)^3^ in the recipient.

Median (range) time to last follow-up was 9 y (1.8 - 28 y). No recipients died with a functioning graft during the follow-up period and 61 (76%) grafts failed by last follow-up. Graft survival by complement defect is shown in Figure 3A. There were insufficient data to control for differences between complement defect groups so statistical comparisons were not performed. Overall, 41 of the 61 grafts that failed had posttransplant TMA (**Table S4, SDC**, http://links.lww.com/TP/C577).

Recipients with pathological variants in *CFH* had high levels of early graft loss with cumulative 1- and 3-y graft survival of 42% and 35%, respectively. In those with VUS, cumulative 1- and 3-y graft survivals were 58% and 50%, respectively. Of the 4 transplants in recipients with pathological variants in CD46, 1 graft failed 10-mo posttransplant from hypertensive donor disease. The diagnosis of donor-derived hypertensive disease in this case is based on review of 3 transplant biopsies carried out between 8 d and 3 mo posttransplantation. Arteriolar hyalinosis with relative glomerular preservation persisted from the initial biopsy and the kidney never functioned well.

Graft survival (Figure 3B) was assessed by recipient risk of relapse (Table 1). In recipients at low (n = 4), medium (n = 21), and high (n = 55) risk of aHUS recurrence, cumulative 6-mo graft survival was 100%, 81%, and 53%, respectively, supporting the KDIGO-advised stratification.^3^

## DISCUSSION

The UK experience strongly supports the use of prophylactic eculizumab treatment at the time of transplantation to improve graft survival in patients with aHUS at medium or high risk of disease recurrence. By preventing aHUS recurrence, eculizumab treatment improved graft survival from 61% to 89% at 3 y, in line with UK outcomes for all causes of ESKD. For the decade 2001-2011, 3-y graft survival for UK adult kidney transplant recipients was 89.5% to 93.9%, depending on donor type and our prophylactic treatment cohort included predominantly deceased donor kidneys.^41^ Transplantation has therefore become a viable and successful option for patients with aHUS reaching ESKD. These findings are in agreement with those reported by Zuber et al,^17^ thus providing further evidence for the current KDIGO approach to transplant care in aHUS.^3^

It was noted that 5 recipients (13%) in the treatment cohort died with a functioning graft compared with none in the control cohort (*P* = 0.057). It is difficult to make a comparison between these death rates given the high rate of early graft failure in the control cohort and that data on patient survival after graft failure were not collected. At 2 y posttransplant, only 61% of grafts in the control cohort continued to function compared with 92% in the treatment cohort and 80% of recipients that died with a functioning graft did so after this time point. As such, the lower death rate with a functioning graft in the control cohort could reflect the low rate of graft survival rather than reduced patient mortality.

NHSBT report 14% of DCD kidney recipients and 11% of DBD kidney recipients die within 5 y of their first kidney transplant.^25^ Data from New Zealand and Australia looking at death in those with a functioning graft found the risk of death 5 y after first kidney transplantation to be 1.9 per 100 patient years. In our cohort, there were 2 deaths in 24 first transplant recipients with a total follow-up of 105 y so not dissimilar to these results.^42^

Eculizumab has been shown to be safe in patients with paroxysmal nocturnal hematuria^43^ and in aHUS.^44^ However, there remains a theoretical concern that the combination of eculizumab with the immunosuppression burden of transplantation could increase mortality.

The findings of retrospective studies are critical to informing the management of kidney transplant recipients with this very rare disease. Our study represents the second report of outcomes from a multicenter patient cohort.^17^ Collectively, these reports demonstrate the transformation in renal graft outcomes that prophylactic eculizumab treatment offers selected patients with aHUS.

We recommend initiating eculizumab treatment prophylactically at the time of transplantation, rather than reactively at the time of aHUS recurrence posttransplant. In part, this is based on the rapidity of disease recurrence demonstrated in our control cohort with graft loss in the first 3 mo exceeding 20%. Following transplantation, the graft is exposed to numerous insults leading to endothelial stress, which may explain the high risk of early recurrence. aHUS in this setting will inevitably perpetuate endothelial injury with the potential for irreversible graft injury.

A concern with treating at the time of disease recurrence is the potential for delay between relapse and eculizumab initiation, particularly when outside the closely monitored early posttransplant period. Patients with kidney transplants can take longer to reach complete TMA response with eculizumab and experience less improvement in renal function compared with those treated for native kidney disease.^14^

We have not investigated the relative effectiveness of reactive and prophylactic treatment in our study. Zuber et al,^17^ found better graft survival with prophylactic versus reactive treatment. In contrast, findings from The Netherlands suggest reactive treatment is possible when endothelial injury can be minimized through use of living donor kidneys, low dose tacrolimus, and strict blood pressure control. Using this protocol, Duineveld et al,^45^ describe successful kidney transplantation without prophylactic eculizumab in 17 recipients at high risk of aHUS recurrence.^45^

Although the Global aHUS registry data showed no reduction in graft loss when eculizumab was initiated prophylactically compared with reactively posttransplant, 2-y eGFR was noted to be better in the prophylactically treated group. However, the groups were not clearly defined, those with *CD46* pathogenic variants were included in the prophylactic treatment cohort and the trigger for eculizumab administration was unclear.^46,47^

Pathogenic variant status is recognized to influence the risk of relapse after renal transplantation in aHUS^8^ and is key to the KDIGO approach to stratification.^3^ We demonstrate a similar pattern to that observed previously with higher risk of recurrence suggested for *CFH* pathogenic variants and relapse being rare with *CD46* variants.^46^ Our data showed poor graft outcomes for those with VUS supporting their classification as medium risk and suggesting some variants may be functionally important.

Given the significant cost and infection risk of eculizumab treatment, the optimal duration of treatment is being investigated. STOPECU (NCT02574403) demonstrated a 23% relapse rate in native kidney disease after eculizumab withdrawal.^48^ Two further trials of eculizumab withdrawal are ongoing (SETSaHUS [ISRCTN17503205], CUREiHUS [Dutch Trial Register NTR5988/NL5833]) and will ultimately determine whether eculizumab withdrawal is safe, in whom it should be attempted, and with what surveillance. Further work is needed to stratify risk of disease recurrence posttransplantation to inform decisions regarding treatment duration and more detailed variant analysis is likely to contribute to this.

In summary, our study demonstrates prophylactic eculizumab treatment significantly improves kidney transplant survival in patients with aHUS with at least medium risk of disease recurrence, making it a viable and superior strategy for renal replacement therapy in these patients. We advocate prophylactic eculizumab treatment before transplantation, rather than reactive treatment posttransplant, as the better option based on currently available data.^17^ When other precipitants of endothelial injury can be minimized, a reactive approach could be considered. Ultimately, the numbers studied remain low so this area would benefit from more targeted research.

## ACKNOWLEDGMENTS

UK aHUS Transplant Consortium: Aimun Ahmed, Damien Ashby, Atif Awan, Richard Baker, Sunil Bhandari, Coralie Bingham, Girish Bommayya, David Border, Cormac Breen, Henry Brown, Alison Brown, Paul Carmichael, Paramit Chowdhury, Simon Curran, Rosie Donne, Chris Dudley, Tina Dutt, Kevin Eardley, Elinor Eblamo-Abad, Daniel Gale, Sian Griffin, John Harty, Peter Hewins, Richard Holt, Victoria Ingham, Hannah Kilbride, Ed Kingdon, Robert Lewis, Nicholas Mansfield, Stephen D Marks, Phil Mason, Paul Mead, Muir Morton, Thalakunte Muniraju, Pramod Nagaraja, Neal Padmanabhan, Nicholas Plant, Tara Raftery, Peter Rowe, Alan Salama, Packiam Shenbagaraman, Alison Taylor, Kerry Tomlinson, Nick Torpey, Mark Uniacke, David Walbaum, Michelle Webb, Matt Williams, Alastair Woodman, Sapna Shah, Imran Saif

This work uses data provided by patients and collected by the NHS as part of their care and support and would not have been possible without access to this data. The NIHR recognizes and values the role of patient data, securely accessed, and stored, both in underpinning and leading to improvements in research and care.

The authors would like to thank Specialist Nurses Amanda Watt and Joanne Stout at the National Renal Complement Therapeutics Centre, Newcastle upon Tyne for their contributions to data collection and maintaining the aHUS database.

## Supplementary Material


