# Supplementary material for: Assessing the Impact of Prophylactic Eculizumab on Renal Graft Survival in Atypical Hemolytic Uremic Syndrome
Source: Transplantation. 2023 Mar 31;107(4):994–1003. doi: 10.1097/TP.0000000000004355 (PMC10065821; doi:10.1097/TP.0000000000004355)
Supplement: Supplementary file 2 [file tp-107-994-s002.pdf]

SUPPLEMENTARY TABLES

Table S1. Inclusion and exclusion criteria for diagnosis of aHUS

Inclusion criteria	Exclusion criteria	Tests for exclusion criteria
Triad of microangiopathic haemolytic anaemia, thrombocytopenia and acute kidney injury<br><br>AND/OR<br><br>Renal biopsy showing TMA	TTP	ADAMTS13 >5%
	Shiga toxin associated HUS*	Stool culture<br>PCR for STEC virulence genes in stool Serology
	Drug-induced TMA	
	Infection (HIV, Streptococcus pneumonia)	HIV<br>T antigen<br>Urinary pneumococcal antigen
	Transplantation (bone marrow, liver, lung, cardiac)	
	Cobalamin deficiency	Plasma homocysteine levels<br>Plasma and urine methylmalonic acid levels<br>MMACHC variant screening
	Systemic lupus erythematosus	dsDNA
	Antiphospholipid antibody syndrome	Antiphospholipid antibodies
	Scleroderma	Antinuclear antibodies<br>Anti-centromere antibodies<br>Anti-scl-70
	Glucose 6 Phosphate Dehydrogenase (G6PD) deficiency	G6PD activity
	Evidence of consumptive coagulopathy	Coagulation Screen Fibrinogen
	Malignant hypertension	Electrocardiogram<br>Echocardiogram
	Plasma cell dyscrasias	Serum/urine electrophoresis<br>Serum free light chains
	Pregnancy with evidence of preeclampsia, HELLP syndrome or TTP	Pregnancy test

The presence of a positive results in a test used to identify an exclusion criteria would go against the diagnosis of aHUS. *Unless a confirmed disease-associated genetic variant was also identified (positive for STEC in n=3, all in prophylactic treatment cohort #93, 321,

387). TMA, thrombotic microangiopathy; TTP, thrombotic thrombocytopenic purpura

Table S2. Group specific data collection.

Control cohort	Prophylactic Treatment Cohort
Native and transplant renal biopsy results<br>Donor type<br>Use of plasma exchange	Native and transplant renal biopsy results<br>Donor type<br>Mismatch<br>Use of plasma exchange<br>Immunosuppression<br>Serum creatinine and eGFR at 1 year<br>Reason for eculizumab cessation<br>Meningococcal vaccination timing

Data collected from medical records, where available, for control and prophylactic eculizumab treatment cohorts in addition to the data collected for transplants and recipients from all groups.

Table S3-A: *MMACHC* primers. DMSO used in exon 1

exon	forward primer sequence	reverse primer sequence
1	GAACTACGCATCCCAAGATGC	GTCACATGGACACTGGGCTG
2	CAAAAGTGTGAGGCCTGAAG	ACCTTTGAAGTGGCTCCTAG
3	ACCATGCCTTCCTTCACACC	CACCACTAGTCTTAACACTGC
4A	GGAAGTGAACAGGCCTAGC	TAGGCTTCTCTGAGGGCTG
4B	ACTTACCGGGATGCTGTGAC	CCTGGCCTTTATCTTGGCC

Table S3-B: *THBD* primers. Betaine used in all amplicons

exon	forward primer sequence	reverse primer sequence
1A	CTGTGCCCCTCTGCTCC	TGGTGTTGTTGTCTCCCGTA
1B	TCATTTCCTTGCTACTGAACG	CACGCTGCAGTCCCAAG
1C	GCTCCCCTCGGCTTACAG	CGTCCACCAGGTCGTAGTTAG
1D	ATACTGGAGCCCAGTCCGTG	GTCACAGTCGGTGCCAATG
1E	GCACGGACATCGACGAG	TTTGGTAGCAAAGCTGGGG

Table S3-C: *VTN* primers. DMSO used in exon 1-2

exon	forward primer sequence	reverse primer sequence
1-2	CTTCTCCAGTGCCCTCCTTC	CATAGTGAACACATCCCCGC
3	AAGGTGTGTTCAGAGCCCAG	GGAGGAGATGGTGTGAGAGC
4	AAGAACGGTTCCCTCTTTGC	CCTGGAGTCTTGGGGCTG
5	AGTGAACCTGGACCTGGG	CCAGAGGCTGTTGAAGTTAGG
6	AGATCCTAACTTCAACAGCCTCT	TCGATAGCTCCACAACCACA
7	GGGGAAGGGAATTGGACTGA	CTGTGGTCACTACTTGCAGG
8	GTTTTCCTTGCTGTCCCTGG	TTAAACTCGGGGCTAAGGGA

Genomic DNA was prepared from peripheral blood according to standard procedures.

Genomic DNA was amplified by PCR using intronic oligonucleotides for MMACHC (Table 2A), THBD (Table 2B) and VTN (Table 2C). The PCR cycling conditions were: 1 cycle of: 95ºC for 5 min; 32 cycles (94ºC for 1 min, 60ºC for 1 min, 72ºC for 1 min); 1 cycle of: 72ºC for 5 min for all amplicons with the exception of THBD amplicons 1B – 1E where an annealing temp of 55ºC was used. All primers contained a tag for sequencing (Tag for forward primer – GTAGCGCGACGGCCAGT; Tag for reverse primer – CAGGGCGCAGCGATGAC).

Mutation screening was carried out by fluorescent sequencing (ABI) and analysis using Mutation Surveyor software (v4.0.8).

Table S4. Graft outcome in cohort not treated with eculizumab.

	Complement defect					
	**VUS** n=12	**Pathological variant**				**anti -FH** n=5
		**CFH** n=43	**CFI** n=5	**C3** n=11	**CD46** n=4	
**Graft status**						
Functioning	2	9	1	2	3	2
Failed	10	34	4	9	1	3
**Cause of graft failure**						
post-transplant TMA	4	27	3	7	0	0
Rejection	2	1	1	1	0	3
Other	4 chronic allograft dysfunction, unclear cause despite investigation, non-viable infarcted kidney, graft thrombosis	2 transplant glomerulo-pathy, cortical rupture, 4 where cause of graft failure not available	0	1 severe chronic tubule-interstitial damage and possible infection	1 hypertensive donor disease	0

Kidney graft outcome in those with atypical haemolytic uraemic syndrome (aHUS) not treated with eculizumab for duration of graft. If the graft failed, cause of graft failure is given where known. Transplants are grouped by complement ,defect in recipient by detection of variant of uncertain significance (VUS) or presence of pathological variant in complement factor H (CFH), complement factor I (CFI), C3 or membrane cofactor protein (CD46) genes, or presence of autoantibodies to factor H (anti-FH). If autoantibodies to factor H were detected concomitantly with a genetic variant, the group was assigned as anti-FH if it was VUS but by the affected gene instead if it was a pathological variant. Therefore, two grafts are grouped by pathological CFH variants when recipients also had anti-FH antibodies detected and 3 grafts in recipients with VUS are grouped as anti-FH due to detection of antibodies. No recipients had identified pathological variants in non-complement pathway genes

associated with aHUS. Number (n) of grafts in each group is detailed. TMA, thrombotic microangiopathy

SUPPLEMENTARY FIGURES

Figure S1. Death-censored renal graft survival without eculizumab treatment by year of transplantation.

Kaplan Meier analysis of graft survival in those at medium and high risk of atypical haemolytic uraemic syndrome recurrence who did not receive eculizumab treatment. Grafts are grouped by year of transplantation to examine for era effect. Survival is censored for patient death with a functioning graft and for functioning graft at last follow up. Numbers at risk in each group at 12 monthly time points are detailed below the graph.

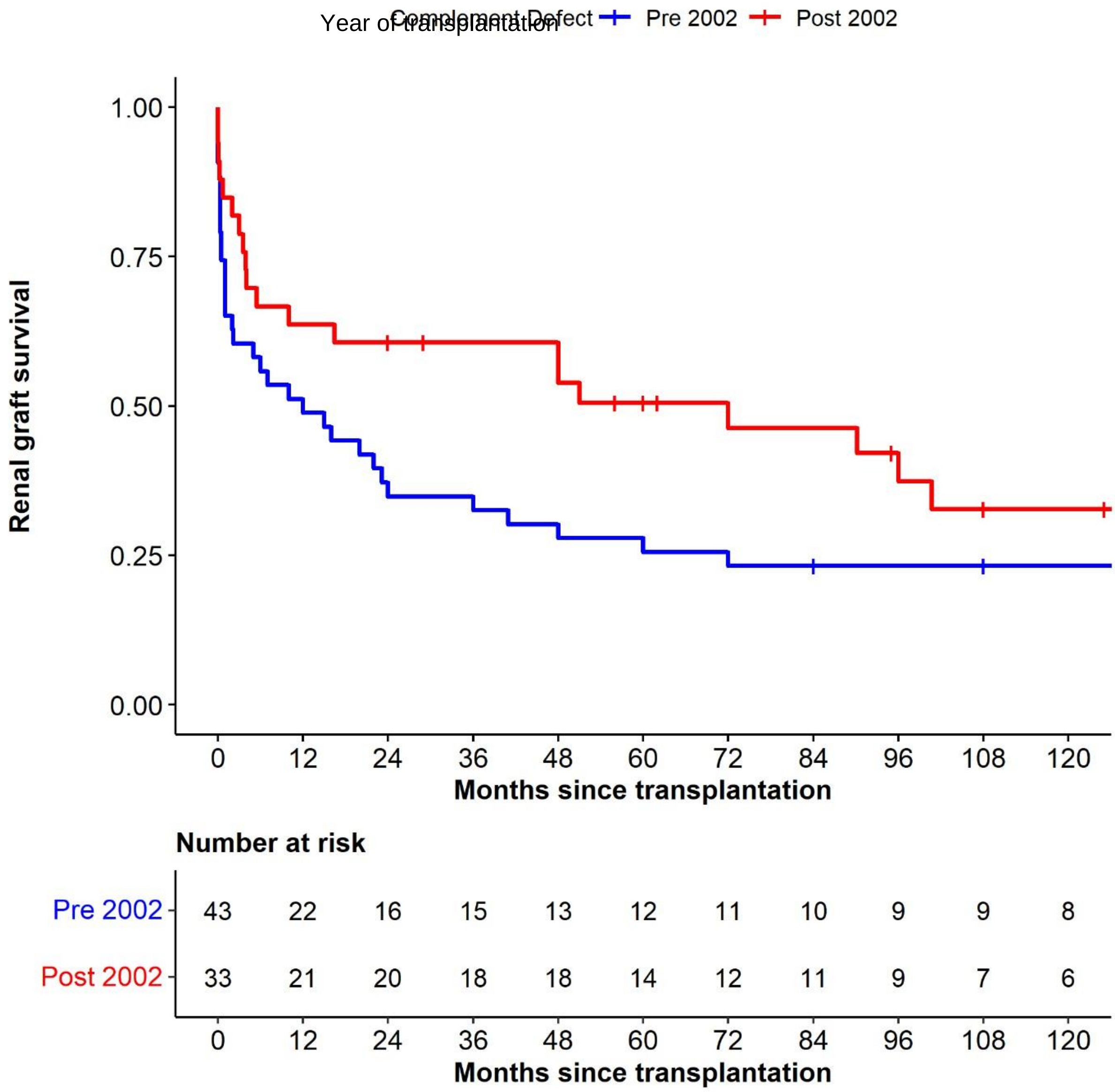

Year of transplantation
Complement Defect
Pre 2002
Post 2002
Renal graft survival
1.00
0.75
0.50
0.25
0.00
0 12 24 36 48 60 72 84 96 108 120
Months since transplantation
Number at risk
Pre 2002 43 22 16 15 13 12 11 10 9 9 8
Post 2002 33 21 20 18 18 14 12 11 9 7 6
0 12 24 36 48 60 72 84 96 108 120
Months since transplantation
